# Robot-Assisted Gait Training Combined with Conventional Physiotherapy in Postoperative Patients with Diplegic Cerebral Palsy: A Pilot Single Cohort Observational Study

**DOI:** 10.3390/s26051438

**Published:** 2026-02-25

**Authors:** Anna Falivene, Emilia Biffi, Luca Emanuele Molteni, Cristina Maghini, Rossella Cima, Roberta Morganti, Eleonora Diella

**Affiliations:** Scientific Institute IRCCS Eugenio Medea, 23842 Bosisio Parini, Italy; anna.falivene@lanostrafamiglia.it (A.F.); emilia.biffi@lanostrafamiglia.it (E.B.); luca.molteni@lanostrafamiglia.it (L.E.M.); cristina.maghini@lanostrafamiglia.it (C.M.); rossella.cima@lanostrafamiglia.it (R.C.); roberta.morganti@lanostrafamiglia.it (R.M.)

**Keywords:** cerebral palsy, robotic training, postoperative patients, neurorehabilitation

## Abstract

Background: Cerebral palsy (CP) is the most common cause of disability in developmental age, affecting motor and postural skills. With growth, lower-limb orthopedic surgery often becomes necessary. Post-surgical walking rehabilitation programs generally involve conventional therapy with only limited evidence on the use of robot-assisted gait training (RAGT). The aim of the present pilot study is to assess the feasibility and the preliminary functional outcomes of an intensive 3-week rehabilitation of 15 sessions with Lokomat combined with 15 sessions of conventional physiotherapy. Methods: In total, 27 patients with diplegic cerebral palsy who underwent orthopedic surgery were recruited. Outcomes collected: the 6 min walking test (primary outcome), the Gross Motor Function Measure-88, the Gillette Functional Assessment Questionnaire, 3D gait analysis, and spasticity and force metrics of the lower limbs. Paired statistical tests were used to assess pre–post changes. Results: A pre–post statistically significant improvement was observed in gait endurance in the 6MWT (Δ = 28.56 ± 34.28 m; *p* < 0.001) and in gross motor functional skills. Gait parameters showed some functional and structural improvements, and joint stiffness was reduced in some measures. Conclusions: This combined rehabilitative approach seems to be promising in postoperative patients with CP. Future studies, involving a control group and larger sample size, are needed to generalize our results.

## 1. Introduction

Cerebral palsy (CP) is the most common cause of disability in the developmental age caused by a non-progressive brain lesion that may occur during the prenatal, perinatal or early postnatal period, altering not only muscle tone, motor and postural skills but also cognitive and behavioral aspects [1,2]. Spastic diplegia is a form of CP that affects mainly the lower limbs; thus, gait quality and efficiency are frequently impaired, leading to progressive musculoskeletal deformities (e.g., hip and knee flexor contractures, equinus foot) that compromise function and quality of life [3,4,5]. Such deformities worsen with age, becoming increasingly disabling and making orthopedic surgery (OS) essential.

OS plays a key role in correcting osteoarticular deformities and improving muscle length, thereby enhancing mobility and preventing long-term complications. Common surgical procedures include single-level or multi-level musculo-tendinous unit release, tendon transfers, osteotomies, and arthrodesis [6]. Post-surgical worsening in walking ability and balance, due to pain, immobilization and muscle weakness, is common [7], requiring intensive postoperative rehabilitative programs. Lower-limb loss of strength is reported 6 months after surgery in comparison with pre-operatory values, and strength training seems to be effective in functional recovery [8,9]; however, the literature on rehabilitative approaches in post-OS CP patients is extremely limited.

Over the past several decades, robotic systems have emerged as rehabilitative tools capable of delivering early rehabilitation programs characterized by high-intensity, repetitive, task-specific and interactive training, thereby enhancing neuroplasticity [10,11].

Moreover, the use of exergames and virtual reality paradigms, together with the possibility of adapting task difficulties to patients’ abilities, help strengthen patients’ engagement [2], which is a key factor in achieving positive rehabilitation outcomes [12].

Several studies have examined the efficacy of RAGT in children with CP. Although RAGT appears to be a valuable complement to conventional physical therapy [13,14], current evidence remains inconsistent, mainly due to variabilities in treatment duration, intensity and frequency [15]. Furthermore, to our knowledge, only two studies have analyzed the effectiveness of rehabilitation with robotic technologies in postoperative patients with CP: Mataki et al. [16] and Kuroda and colleagues [17] assessed the effect of the Hybrid Assistive Limb^®^ exoskeleton (CYBERDYNE, Tsukuba, Japan) on one patient and six participants respectively, demonstrating an overall improvement in patients’ performance. However, evidence of RAGT efficacy in post-surgical CP is still limited and based on small-sample case studies.

Thus, the aim of the present study was to assess the feasibility and the preliminary functional outcomes of an intensive 3-week rehabilitation program combining RAGT and CT in patients with diplegic CP following orthopedic surgery. We hypothesized that this training protocol would lead to improvements in both functional abilities and gait-related outcomes. Specifically, we expected results to be consistent with the previous literature on post-surgical CP patients and slightly superior to those reported in studies adopting an intensive rehabilitation approach in non-surgical CP populations, due to the greater potential for functional modification following surgery. This assumption was supported by the study of Gorton and colleagues [18], whose prospective RCT compared impairment and functional outcomes in postoperative CP patients with a non-surgical group, reporting significant improvements in the postoperative group at the one-year follow-up.

## 2. Materials and Methods

### 2.1. Design

**Figure 1:**
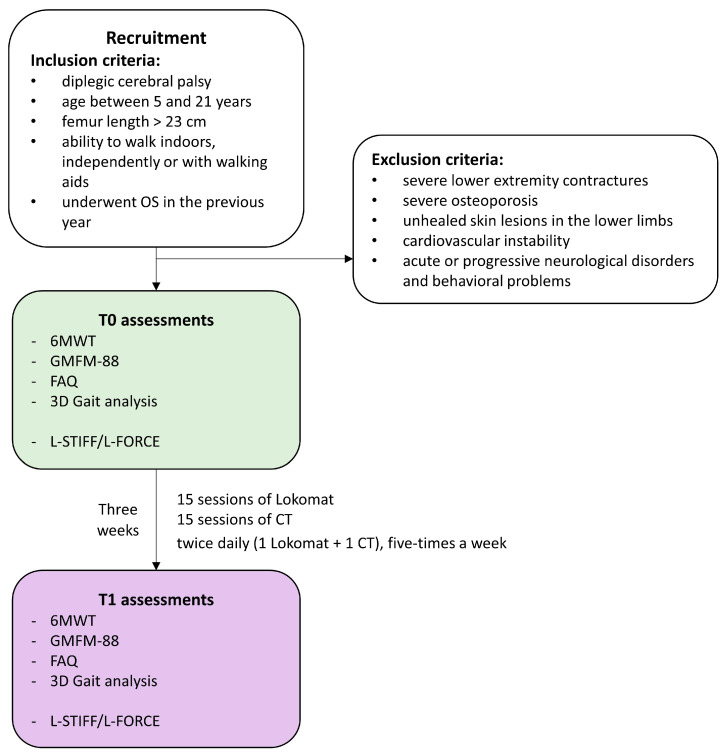
Flowchart of the study. OS: Orthopedic Surgery, CT: Conventional therapy, 6MWT: 6 min walking test, GMFM-88: Gross Motor Function Measure-88, FAQ: Gillette Functional Assessment Questionnaire. L-STIFF and L-FORCE: stiffness and isometric voluntary force measured by the Lokomat.

This is a retrospective single-cohort observational study conducted following STROBE guidelines in Supplementary Table S1 [19]. Given its observational and retrospective design, the study was conceived as exploratory and hypothesis-generating, with the primary aim of assessing the feasibility and preliminary functional outcomes of the proposed rehabilitation program, rather than establishing causal relationships. The study flowchart is represented in Figure 1.

### 2.2. Participants

The present study includes patients diagnosed with diplegic CP, recruited in the period between February 2017 and September 2023 at the Scientific Institute Eugenio Medea in Bosisio Parini, Italy.

Inclusion criteria were: age between 5 and 21 years; femur length > 23 cm; ability to walk indoors, independently or with walking aids; and OS performed in the previous year. Exclusion criteria were: severe lower extremity contractures; severe osteoporosis; unhealed skin lesions in the lower limbs; cardiovascular instability; and acute or progressive neurological disorders and behavioral problems.

### 2.3. Intervention

The rehabilitation program consisted of 30 rehabilitation sessions (15 RAGT sessions with the Lokomat combined with 15 CT sessions). Sessions were performed twice daily (1 Lokomat + 1 CT), five-times per week, over three consecutive weeks. Each session lasted 45 min. Both RAGT and CT were delivered by trained and expert physiotherapists.

The Lokomat Pro system (Hocoma AG, Volketswil, Switzerland) is a Class IIa medical device that combines a body weight support (BWS) system with a treadmill. The hip and knee joints of the orthosis are actuated by two drives per leg and controlled by a real-time system that adjusts joint angles to replicate physiological gait patterns.

According to the manufacturer’s guidelines, an initial short therapy session (approximately 15 min) was performed with the BWS set to around 50% of the patient’s body weight. Training started with maximum robotic assistance (i.e., guidance force set at 100%) and a comfortable walking speed. Subsequent Lokomat sessions lasted about 45 min with 30 min of active training, during which both the BWS and guidance force were gradually decreased and walking speed was increased, based on the individual patient’s tolerance and performance. Body weight support was reduced as much as was safely tolerated, while guidance force was decreased as long as the patient maintained a physiological and symmetrical gait pattern. Walking speed was increased in increments of 0.1 km/h when patients were able to actively participate and preserve gait quality. Session-by-session reports were systematically stored to monitor patient progress and guide treatment. Throughout the rehabilitation program, individualized training parameters and exercise characteristics were continuously adjusted to challenge the patient appropriately, promote engagement, and support functional recovery.

Conventional therapy sessions lasted 45 min and followed a multidimensional approach aimed at improving gait capacity. First, stretching programs were administered, targeting the hip and knee flexors, hip adductors, knee extensors and ankle plantar flexors to reduce muscle spasticity and increase joint mobility. Second, selective and functional strength training was performed to enhance muscle control at the hip and pelvis level. Finally, the sessions focused on specific gait training based on motor-learning principles emphasizing repetitive task-specific practice and appropriate feedback. Training included weight shifting, static and dynamic balance exercises, walking with different patterns (e.g., sideways walking, backward walking, stair climbing) and dual task training combining motor tasks [20].

### 2.4. Outcome Measures

Patients’ motor skill levels were defined before treatment through the Gross Motor Function Classification System (GMFCS) [21]. To evaluate the effect of the intensive combined rehabilitation training, a motor assessment was performed before (T0) and at the end of the treatment (T1), including the outcome measures listed and described in detail in Table 1. All the assessments described below were delivered independently by trained and expert physiotherapists.

We selected the 6 min walking test (6MWT) as the primary outcome of the study to assess patients’ gait endurance [22] during self-paced walking within 6 min through the hospital corridors. Verbal standardized instructions were given to the patient during the test, which included walking at his/her maximal (but comfortable) speed, turning 180 degrees every 25 m and covering as much distance as possible within the 6 min. The outcome of this test is thus the distance covered during this amount of time.

Secondary outcomes were the Gross Motor Function Measure-88 (GMFM-88), the Gillette Functional Assessment Questionnaire (FAQ) and 3D-Gait Analysis (3DGA), and lower-limb stiffness and isometric voluntary force.

GMFM-88 evaluates gross motor skills in patients with CP and is validated for use in individuals aged from 6 months up to 18 years old. It includes 88 items, divided into 5 dimensions [23] (i.e., A: lying and rolling; B: sitting; C: crawling and kneeling; D: standing; E: walking, running, and jumping). The score for each dimension (expressed both as an absolute value and as a percentage) was then calculated. Additionally, the global score was computed as the sum of the scores of the 5 dimensions.

The FAQ was administered to the patients, or their parents when needed, to assess functional autonomy in walking during daily life on a 10-level classification [24]. Level 1 represents the subject’s inability to take any steps, while level 10 represents a condition in which they are able to walk and run, even on uneven or rough terrain, without difficulty or assistance.

3DGA provides a quantitative analysis of gait movement. The laboratory at the IRCCS E.Medea is equipped with an optoelectronic system (Elite, BTS Bioengineering, Milan, Italy), with eight infrared cameras (sampling rate: 100 Hz), and four force plates (P6000; BTS Bioengineering, Milan, Italy) embedded in the floor (sampling rate: 200 Hz). Twenty-two passive spherical markers were placed on patients’ body according to the Davis protocol [25]. Patients were asked to walk barefoot along a 10 m walkway at their preferred speed, performing an average of five repetitions. The most representative trial was then selected by an expert physiotherapist and processed with the BTS SmartAnalyzer software 1.10.0470 (BTS Bioengineering, Milano, Italy) for data extraction and further analyses. To qualitatively compare each gait parameter with the corresponding normative curve, mean and standard deviation values provided by the BTS Smart Clinic software for healthy children were considered and reported. Additionally, to assess deviation from normality for kinematic gait features, the Gait Variable Scores (GVSs) and the Gait Profile Score (GPS) were derived, with higher values representing a larger deviation and a more abnormal gait [26].

Finally, at the beginning of the second RAGT session and of the last session, patients’ lower-limb (i.e., hip and knee flexors and extensors) stiffness (L-STIFF) and isometric voluntary force (L-FORCE) were measured directly through the Lokomat system, which is equipped with force transducers. The spasticity of the hip and knee flexors and extensors was measured by means of muscle mechanical stiffness during controlled passive movements of the legs, performed at three different velocities (22.5°/s, 45°/s, and 90°/s; peak angular velocity). Moreover, the isometric force produced by the patient in the hip and knee was measured during flexion–extension movements. For clarity of presentation, these measurements will also be referred to as belonging to T0 and T1 time points.

### 2.5. Statistical Analysis

Patient sample size was estimated with G*Power 3.1.9.4, setting the significance level to 0.05 and the power to 0.9. As for the present pilot study, a medium-large effect size (ES = 0.65) was hypothesized.

Data normality was assessed with the Shapiro–Wilk test. Based on data distribution, a Student *t*-test or a Wilcoxon signed-rank test was used to investigate any statistical differences between the outcomes at T0 and T1. Effect sizes were estimated using test-specific metrics, with Cohen’s d reported for *t*-tests and r for Wilcoxon tests. Possible outliers were removed prior to statistical analysis.

For each patient, the severity of the condition on both sides was classified as either more affected or less affected based on clinical evaluations. For the features that were extracted for both the right and left legs (i.e., most of gait parameters, L-STIFF, and L-FORCE) before any statistical analysis, data were then re-aggregated based on clinical evaluations, considering the most affected side (hereafter: Impaired, I) and the less impaired one (hereafter: Less Impaired, LI), to better identify potential differences in improvement.

For all statistical tests, a *p* value correction for multiple comparisons was performed applying the Bonferroni–Holm method. This correction was performed within predefined subcategories of parameters rather than across all variables simultaneously due to the exploratory nature of the present study. More specifically, we applied the correction to the GMFM features, to the L-STIFF Lokomat metrics, and to the L-FORCE metrics, both considering hip and knee-related metrics separately. Nevertheless, given the exploratory nature of the present study and in line with previous studies in the literature investigating gait parameters in pediatric patients with CP [27,28,29,30], we did not apply multiple-comparison correction to gait-related parameters. Significance level was then set at adjusted *p* < 0.05.

All statistical analysis was performed with SPSS statistics (version 21, Chicago, IL, USA) and MATLAB (R2023a, The Mathworks, Natick, MA, USA).

The effect of the treatment was also assessed in relation to minimal clinically important difference (MCID) values for children with CP, when available in the literature, for the significant outcomes. For the primary outcome, an MCID equal to 15 m was set as an intermediate value of the range of 6–23 m defined by Storm et al. for CP patients [31]; similarly for the GMFM-88 scores, an MCID of 1.5% was considered from the range of 0.1–3% suggested by [31]; for the dimensions GMFM-D and GMFM-E, values of 1.2% and 1.6% were imposed respectively [32]. For gait kinematics in the sagittal plane, we set the MCID to 5°, as proposed in [33].

## 3. Results

### 3.1. Participants

The data of 27 participants (Table 2) affected by diplegic CP, who underwent orthopedic surgery in the year prior to recruitment, were collected for the present study.

Table 3 presents mean ranges of the Lokomat setting parameters used during patient intervention, divided per GMFCS group levels.

### 3.2. Analysis

#### 3.2.1. Clinical Assessment

Clinical evaluations were collected for all participants, except for the GMFM-88 assessed for 22 participants (minors under 18 years). Table 4 reports the descriptive statistics of the clinical outcome measures, along with statistical results.

**Figure 2:**
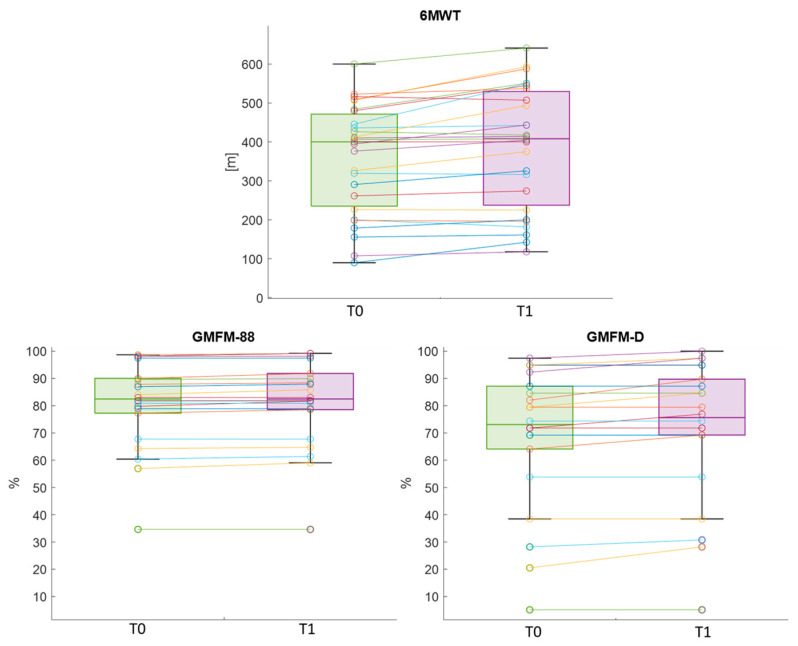
Boxplots reporting significant differences between T0 and T1 in functional outcome measures. 6MWT = Six-Minute Walking Test; GMFM = Gross Motor Function Measure.

After treatment, the primary outcome of the study presented a statistically significant difference, with an increased distance walked in the 6MWT (Cohen’s *d* = 0.83). Similarly, the GMFM-88 and GMFM-D dimension showed significant improvements, with an effect size equal to 0.68 and 0.58 respectively (Figure 2). No pre–post differences in FAQ values were found.

For the primary outcome, 74% of participants improved in walked distance, with 48% showing clinically relevant improvements, whereas just one subject experienced a clinically relevant decline in performance. The percentage of patients with improved gross motor abilities in the GMFM-88 and GMFM-D were 59% and 41% respectively. Specifically, 18%, 41% and 13.6% of patients had clinically relevant changes respectively. Nobody experienced a decline in his/her gross motor abilities.

#### 3.2.2. Gait Parameters

Gait analysis was performed for all participants except those classified at level GMFCS IV. In these cases, the severe limitations in autonomous walking required the use of substantial assistive devices that were not compatible with the standardized gait analysis setup. As a result, it was not possible to obtain reliable or reproducible gait patterns under laboratory conditions, and these participants were therefore excluded from the quantitative gait analysis.

Below, results related to features on the sagittal plane are presented. Results concerning coronal and transverse planes are presented in Supplementary Table S2.

**Figure 3:**
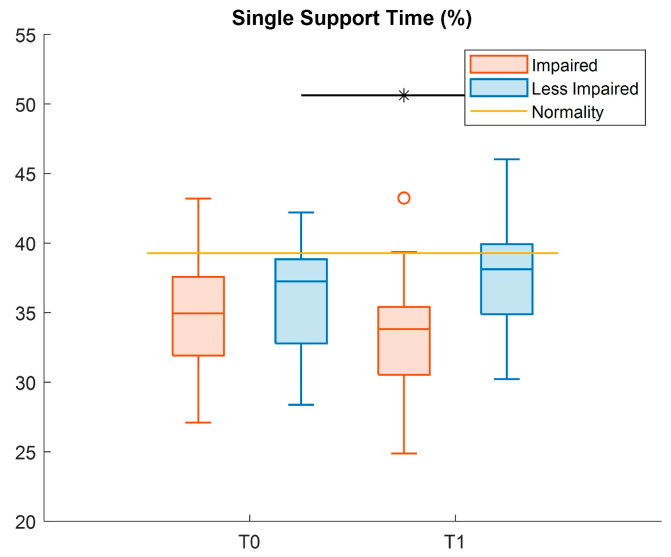
Boxplots reporting significant pre–post differences in the single support time metric for both impaired (red) and less impaired (blue) legs. Black asterisk represents statistically significant differences (*p* < 0.05).

Table 5 reports the descriptive statistics of the spatio-temporal outcome measures, and the results of statistical tests, with only the LI-leg showing a significant increase (Cohen’s *d* = 0.46) for the single support time parameter at T1 (Figure 3).

**Figure 4:**
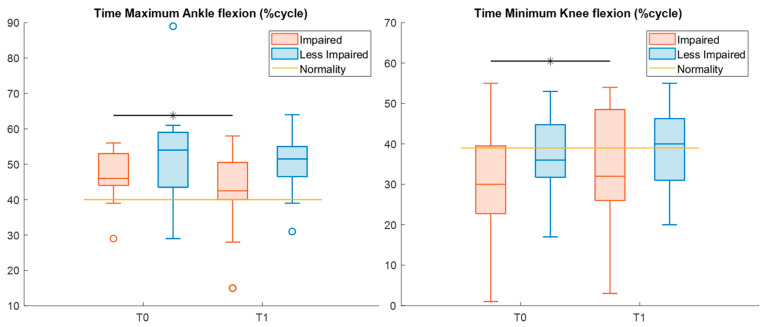
Boxplots reporting significant pre–post differences in ankle and knee kinematic features for both impaired (red) and less impaired (blue) legs. Black asterisks represent statistically significant differences (*p* < 0.05).

Table 6 displays descriptive statistics and the results of the statistical analysis of kinematic features in the sagittal plane. Specifically, from T0 to T1, the time of maximum flexion of the impaired ankle was reduced (Cohen’s *d* = 0.85), whereas the time of minimum flexion of the impaired knee significantly increased (Cohen’s *d* = 0.63), nearing normal values (Figure 4).

**Figure 5:**
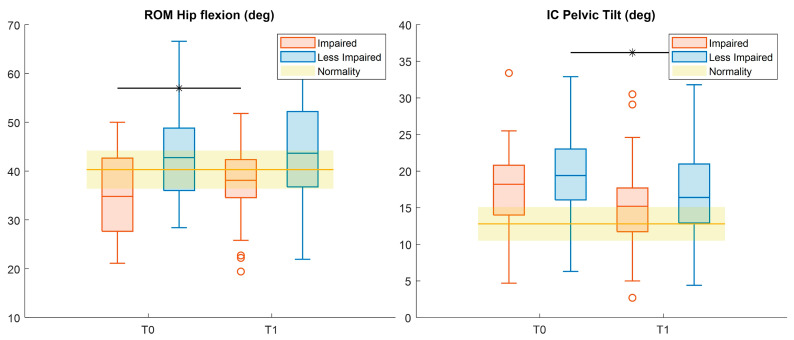
Boxplots reporting significant pre–post differences in: Hip and pelvic kinematic features for both impaired (red) and less impaired (blue) legs. Black asterisks represent statistically significant differences (*p* < 0.05).

Considering hip kinematics (Figure 5), pre–post significant differences were found for the range of motion (ROM) in the impaired leg, with increasing values at T1 (Cohen’s *d* = 0.42). Moreover, the pelvic tilt at initial contact for the LI-leg showed a significant reduction after treatment (Cohen’s *d* = 0.44) (Figure 5). All these changes moved the values toward normality.

**Figure 6:**
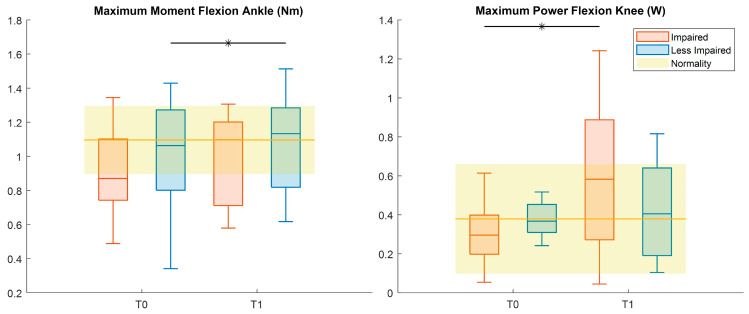
Boxplots reporting significant pre–post differences in 3D gait kinetic features for both impaired (red) and less impaired (blue) legs. Black asterisks represent statistically significant differences (*p* < 0.05).

Kinetics analysis (Table 7, Figure 6) showed an increased peak during LI-ankle flexion (Cohen’s *d* = 0.6) and increased maximum power during flexion of the knee for the impaired leg (Cohen’s *d* = 1).

**Figure 7:**
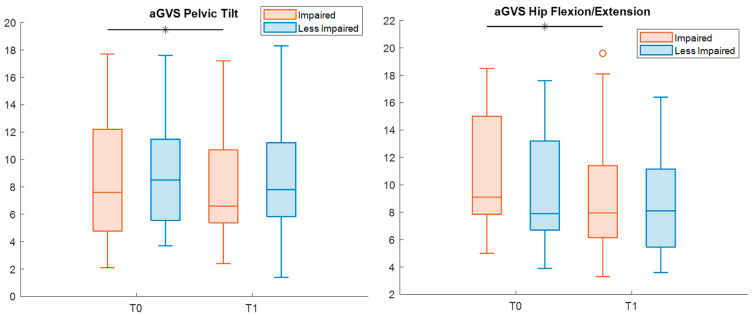
Boxplots reporting significant pre–post differences in Gait Variable Scores (aGVSs) for both impaired (red) and less impaired (blue) legs. Black asterisks represent statistically significant differences (*p* < 0.05).

Finally, when evaluating potential differences from normality, the analysis of the GVS yielded a significant reduction in the deviation in pelvic tilt and hip flexion–extension for impaired legs (effect size r = 1.4 and 0.9, respectively) (Table 8, Figure 7).

Gait pattern improvements were evaluated clinically for significant outcomes, when possible. In particular, the ROM of the hip of the impaired leg increased in 60% of patients, with 24% above MCID. Conversely, 12% of patients showed a reduction in ROMs larger than the MCID.

#### 3.2.3. Lokomat Parameters

**Figure 8:**
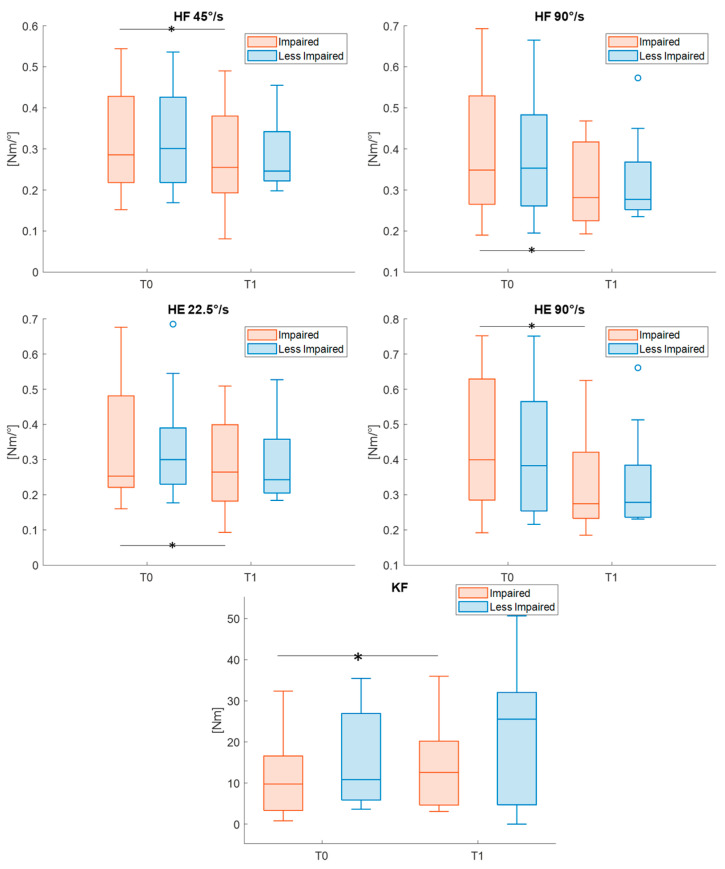
Boxplots reporting pre–post differences in L-STIFF and L-FORCE features for both the impaired and less impaired groups. Asterisks represent statistically significant differences (*p* < 0.05). HF = Hip Flexion; HE = Hip Extension; KF = Knee Flexion.

L-STIFF and L-FORCE metrics were recorded directly by the Lokomat system in 17 participants. Descriptive statistics and statistical results are reported in Table 9. Following treatment, a reduced (all effect sizes > 0.61) L-STIFF of impaired hips (during both flexion and extension) and a statistically increased (Cohen’s *d* = 0.76) L-FORCE metric in the impaired knee during flexion were found. Significant pre–post differences after Bonferroni–Holm correction are highlighted in Figure 8.

## 4. Discussion

The study presents the preliminary results obtained through robotic gait training with the Lokomat system combined with conventional physical therapy in postoperative patients affected by diplegic CP.

The obtained results showed statistically significant improvements in functional performance, both in the primary outcome of the study (i.e., 6MWT), with a moderate percentage of patients presenting a clinically relevant change, and in gross motor abilities.

Comparing our results with the previous literature on RAGT in post-surgical CP patients, we observed a similar trend of improvement in gait endurance and gross motor function to that reported by Kuroda et al. [17]. Specifically, 48% of participants of our study showed clinically relevant improvements in walked distance. Clinically meaningful improvements were also observed in gross motor, with 18% of patients improving in GMFM-88 and 41% in GMFM-D. Despite a more heterogeneous sample in terms of GMFCS levels, type of orthopedic surgery, and time since surgery, the alignment in functional gains suggests that RAGT combined with conventional therapy may support early post-surgical recovery of mobility. These findings should be interpreted as preliminary and hypothesis-generating, given the pilot nature of the study.

In comparison with Beretta and colleagues [34], our post-surgical patients showed an increase in a 6MWT distance of comparable magnitude. Specifically, Beretta and colleagues reported an average improvement of approximately 12%, whereas in our study, the increase was about 8%, despite the training period being one week shorter. This comparison should be interpreted with caution, given differences in patient characteristics, surgical status, and study design, and should be considered a preliminary observation rather than evidence of superiority.

Concerning gait spatio-temporal parameters, we obtained a small but significant increase in the single support time for the LI-leg, as also observed by Wallard et al. who analyzed the effect of 20 RAGT sessions in a group of 14 CP patients [29]. Children with CP are reported to have reduced single support time [35] with respect to typically developing (TD) children, due to muscle weakness and balance impairment [36]. Therefore, a longer single support time can be attributed to an improvement in pelvis and hip control, enhancing weight-bearing capacity and balance.

In joint kinematics, a significantly improved timing of muscle activation was observed following treatment for both ankle and knee joints of the impaired leg, which could be related to a reduction in plantar-flexor spasticity [37,38]. In more detail, following treatment, the peak ankle dorsiflexion occurred, on average, about 5% earlier during the gait cycle, indicating a more physiological activation pattern compared with the baseline [37]. Similarly, the delayed peak of knee extension at T1 showed an improvement in mid-stance knee dynamic control [38].

Considering structural changes, our data showed an increased ROM hip flexion for the impaired limb at T1, which could be considered a promising result given the decreasing trend in ROM hip flexion reported in natural history studies on CP patients [39]. Furthermore, the LI-pelvic tilt at initial contact at T1 exhibited a significant reduction, probably due to an improvement in the pelvic proximal control on the sagittal plane with a reduction in compensatory strategies.

Different from our kinematic data, Wallard et al. found statistically significant changes mainly in the ROM of ankle and knee joints in 14 CP patients who underwent 20 RAGT sessions; no changes were reported for hip and pelvis kinematics [28].

The analysis of kinetic data revealed a slight increase in the LI-ankle flexion peak moment and a higher maximum peak in the impaired-knee power curve over the gait cycle after treatment. Compared with TD peers, patients with CP generate less energy during the stride [40]; therefore, the increase in knee power at T1 may indicate a meaningful improvement in knee flexor strength.

The analysis of deviation from normality showed an improvement only at the hip and pelvic levels. Specifically, the reduction in the GVS of hip flexion–extension at T1 seems to confirm our findings on hip kinematics. In addition, the GVS of pelvic tilt resulted in being lower at T1, supporting our considerations on better pelvic postural control.

Finally, the significant reduction in the L-STIFF parameter, observed at higher velocities (to evoke the stretch reflex), can be associated with a reduction in joint spasticity, as also reported in previous studies on children with CP [41]. In addition, isometric force assessment (i.e., L-FORCE metrics) showed a significant pre–post difference for the impaired knee during flexion, corroborating kinetic data.

This work has some limitations. First of all, the exploratory design of the study, together with the absence of a control group, does not allow for causal inference regarding the efficacy of the proposed combined rehabilitative approach, nor for a broad generalization of the results. Moreover, the participants recruited for the study underwent different types of OS (e.g., osteotomies, musculo-tendinous unit release, tendon transfers) at different body levels, and presented a wide range of motor impairment (GMFCS levels I–IV). This clinical heterogeneity may have influenced the observed outcomes and represents an important source of variability, reflecting the real-world clinical population included in the study. Anticipating treatment administration and collecting larger patient cohorts could be useful to better exploit the modifiability determined by surgery and increase the reliability of our results, allowing the analysis of subgroups with different levels of GMFCS or different types of OS.

In addition, the lack of a follow-up assessment prevents the evaluation of medium- and long-term effects of the intervention. Moreover, a pre-operation assessment, that could have allowed a pre–post-surgery comparison on patient performance, is also missing in the current study. Saleh et al. analyzed the natural postoperative course in CP patients after Achilles’ tendon lengthening, observing significantly increased GMFM scores only after 24 months, while the variation reported between the 6th and 12th month could be considered quite stable [42]. In our study, we already recorded significant differences within 3 weeks of intensive training in the one year-period after surgery. Therefore, although it is not possible to draw conclusions regarding the preoperative period, we can assume that these improvements may at least be partly due to RAGT + CT treatment. Despite these limitations, this study represents a pilot, hypothesis-generating study aimed at exploring the feasibility and preliminary functional outcomes of an intensive combined robotic and conventional rehabilitation program in post-surgical CP patients, a field that has been only marginally investigated to date. The present findings highlight the potential interest of such an approach, while underscoring the need for more robust study designs.

In the future, it would be interesting to investigate the efficacy of an earlier intensive RAGT intervention in the post-surgical phase (2–3 months after OS) and/or a longer period of training (4–6 weeks) to evaluate whether better results can be achieved. Grecco et al., reported favorable functional results in 15 post-surgery CP patients who received CT approximately 2 months after surgery [43].

Future research should include prospective randomized controlled trials with larger and more homogeneous samples, the inclusion of appropriate control groups, and longitudinal follow-up assessments with multiple pre- and post-surgery time points to better characterize treatment effects over time. A recent study by Carman and colleagues highlights the importance of individualized goal setting and patient-focused outcomes in patients with CP undergoing OS [44]. In this context, patient-reported outcome measures (PROMs), such as the Canadian Occupational Performance Measure (COPM) and Goal Attainment Scaling (GAS) may be particularly valuable for evaluating post-surgical outcomes and determining whether surgical goals have been achieved. Additionally, integrating gait analysis with electromyography could provide further insights into changes in muscle activation patterns and support a more comprehensive interpretation of functional gait adaptations.

## 5. Conclusions

This is one of the few studies investigating rehabilitation treatment in post-surgical CP patients. The intensive and combined approach (RAGT + CT) proved to be a feasible and potentially promising intervention for this population. Functional outcomes showed preliminary improvements in gait endurance, which appear advantageous compared with those reported in the existing literature. However, given the pilot nature of the study, these findings should be interpreted with caution, and the efficacy of the proposed program needs to be confirmed through randomized controlled trials with larger samples and a long-term follow-up.

## Figures and Tables

**Table 1:** Outcome measures collected at the beginning and at the end of the intensive rehabilitation treatment. 6MWT: Six-Minute walking test; GMFM-88 = Gross Motor Function Measure-88; FAQ = Functional Assessment Questionnaire; ROM = Range of Motion; IC = Initial contact; GVS = Gait Variable Score; GPS = Gait Profile Score.

Outcomes		Description
Clinical evaluations	6MWT	Timed test that measures the distance covered during self-paced walking within 6 min along a standardized 25 m path
	GMFM-88	Assessment tool including 88 items, divided into 5 dimensions: A: Lying and rolling; B: Sitting; C: Crawling and kneeling; D: Standing; E: Walking, running, and jumping.
	FAQ	Assessment of functional autonomy in walking during daily life on a 10-level classification
3D- Gait Analysis	Spatio-temporal parameters	walking velocity cadence bilateral stride duration bilateral step length bilateral step width
	Kinematics data	ROM Mean angle IC angle for ankle, knee, hip pelvis and trunk.
	Kinetic data	Moment Power for ankle, knee, and hip.
	GVS	Measure of the distance from the corresponding normative curve: Ankle Dorsiflexion Ankle Intra/Extra Rotation Pelvic Tilt Pelvic Rotation Pelvic Obliquity Hip Flexion/Extension Hip Abduction/Adduction Hip Intra/Extra Rotation Knee Flexion/Extension
	GPS	Summary index derived from three-dimensional kinematic gait analysis that quantifies the overall deviation of an individual’s gait pattern from a healthy reference group. It is computed as the root mean square (RMS) difference between the subject’s joint angle trajectories and normative data across the gait cycle.
Lokomat parameters	L-STIFF	Muscle mechanical stiffness measured as the torque required to obtain controlled passive movements of the legs at three different velocities: hip: 22.5°/s, 45°/s, and 90°/s; peak angular velocity; knees: 30°/s, 60°/s and 120°/s; peak velocity
	L-FORCE	Isometric voluntary force produced in the hip and knee during flexion–extension movements.

**Table 2:** Sample description. Mean (standard deviation—SD) values are reported for age and time after surgery metrics.

Age (Year)	13.93 (3.37)
Gender (males\females)	18\9
GMFCS (level I\II\III\IV)	8\8\9\2
Type of surgery (bone\soft tissues\both)	4\14\9
Time from surgery (months)	6.29 (2.92)

**Table 3:** Mean ranges (minimum–maximum value) of the Lokomat setting parameters divided per GMFCS levels.

GMFCS	Speed Range (m/s)	GF Range (%)	BWS Range (%)
1	(1.51–1.96)	(67–100)	(8.13–35.13)
2	(1.4–1.92)	(74.4–100)	(5.8–40.12)
3	(1.29–1.8)	(82.2–100)	(12.8–41.1)
4	(1–1.45)	(100–100)	(20.5–47)

**Table 4:** Functional measures. Based on the data distribution, median/mean (IQR/SD) values are reported. Bolded texts represent statistically significant differences (adj *p* < 0.05). ^a^ Student *t*-test; ^b^ Wilcoxon signed-rank test.

Outcomes		T0	T1	Adj *p* Value
**6MWT (m)**		**358.47 (139.81)**	**387.04 (155.38)**	**<0.001 ^a^**
FAQ		8 (2)	8 (2)	0.125 ^b^
GMFM (%)	**GMFM-88**	**82.46 (12.76)**	**82.46 (13.27)**	**0** **.006 ^b^**
	A	100 (3.92)	100 (3.92)	1 ^b^
	B	100 (3.33)	100 (3.33)	0.314 ^b^
	C	91.67 (30.95)	92.86 (28.57)	0.177 ^b^
	**D**	**73.08 (23.08)**	**75.64 (20.51)**	**0** **.035 ^b^**
	E	57.32 (29.70)	57.89 (29.45)	0.064 ^a^

**Table 5:** Spatio-temporal 3D gait outcomes. Based on the data distribution, median/mean (IQR/SD) values are reported. Bolded texts represent statistically significant differences (*p* < 0.05). ^a^ Student *t*-test; ^b^ Wilcoxon signed-rank test. I = Impaired; LI = Less Impaired; GDI = Gait Deviation Index.

		T0	T1		
Outcomes	Impairment Level	Mean/Median (SD/IQR)	Mean/Median (SD/IQR)	*p* Value	Normal Value (SD)
velocity (m/s)	-	0.7 (0.29)	0.71 (0.3)	0.641 ^a^	1.2 (0.2)
cadence (step/Min)	-	108.86 (16.92)	106.72 (16.06)	0.403 ^a^	129.6 (8.4)
step width (m)	-	0.15 (0.05)	0.16 (0.06)	0.321 ^a^	0.08 (0.04)
step length (m)	I	0.43 (0.24)	0.45 (0.2)	0.165 ^a^	0.58 (0.06)
	LI	0.4 (0.11)	0.4 (0.14)	0.844 ^a^	
single support time (%)	I	34.69 (4.16)	33.66 (4.28)	0.262 ^a^	39.28 (37.25)
	**LI**	**36.32 (3.86)**	**37.65 (4.11)**	**0.045 ^a^**	
stance (%)	I	63.38 (3.82)	62.48 (4.32)	0.238 ^a^	57.97 (1.93)
	LI	65.57 (4.37)	66.19 (4.38)	0.448 ^a^	
swing (%)	I	36.62 (3.82)	37.52 (4.32)	0.238 ^a^	42.03 (1.93)
	LI	34.43 (4.37)	33.81 (4.38)	0.448 ^a^	
GDI	I	76.64 (7.1)	78.02 (9.75)	0.334 ^a^	-
	LI	79.37 (12.12)	78.84 (10.98)	0.732 ^b^	-

**Table 6:** Kinematic 3D gait outcomes in the sagittal plane. Based on the data distribution, median/mean (IQR/SD) values are reported. Bolded texts represent statistically significant differences (*p* < 0.05). ^a^ Student *t*-test; ^b^ Wilcoxon signed-rank test. I = Impaired; LI = Less Impaired; IC = Initial Contact; ROM = Range of Motion; AF = Ankle Flexion; KF = knee flexion; HF = hip flexion.

		T0	T1		
Outcome	Impairment Level	Mean/Median (SD/IQR)	Mean/Median (SD/IQR)	*p* Value	Normal Value (SD)
IC AF (deg)	I	−7.5 (5.52)	−7.66 (6.6)	0.879 ^a^	−1.9 (3.9)
	LI	−2.91 (6.64)	−3.21 (6.7)	0.757 ^a^	
ROM AF- Stance (deg)	I	15.73 (5.06)	16.78 (6.2)	0.252 ^b^	15.2 (3.3)
	LI	16.2 (7.35)	15.7 (4.55)	0.17 ^a^	
ROM AF- Swing (deg)	I	10.63 (4.19)	10.83 (4.82)	0.787 ^a^	10.7 (3.25)
	LI	10.72 (3.89)	11.92 (5.81)	0.164 ^a^	
Time maximum dorsiflexion (%)	**I**	**46 (9)**	**42.5 (10.5)**	**0.008 ^a^**	40
	LI	52.63 (13.91)	50.75 (8.19)	0.55 ^a^	
Time maximum plantarflexion (%)	I	74 (33)	67.5 (6)	0.113 ^b^	64
	LI	69.5 (8)	69.5 (7)	0.878 ^a^	
IC KF (deg)	I	16.09 (7.64)	16.27 (7.25)	0.88 ^a^	9.4 (3.4)
	LI	18.08 (6.3)	17.99 (7.84)	0.938 ^a^	
Time Maximum KF (%)	I	79.28 (6.5)	80.16 (6.25)	0.24 ^a^	72
	LI	80.48 (6.05)	81.12 (7)	0.525 ^a^	
Time Minimum KF (%)	**I**	**30.24 (14.42)**	**35.81 (14.25)**	**0.009** **^a^**	39
	LI	37.33 (9.56)	38.62 (10.22)	0.504 ^a^	
ROM KF (deg)	I	38.56 (12.98)	37.72 (12.38)	0.531 ^a^	50 (4)
	LI	46.98 (13.3)	47.2 (13.97)	0.889 ^a^	
IC HF (deg)	I	40.17 (10.06)	39.32 (8.93)	0.337 ^a^	37.9 (3.1)
	LI	44.01 (7.35)	42.64 (8.91)	0.178 ^a^	
Time Maximum HF (%)	I	90.43 (4.88)	90.36 (6.1)	0.938 ^a^	85
	LI	92.5 (3.16)	93.07 (3.02)	0.365 ^a^	
Time Minimum HF (%)	I	54.8 (3.58)	54.55 (3.83)	0.747 ^b^	51
	LI	55 (6)	55 (4.5)	0.292 ^a^	
ROM HF (deg)	**I**	**35.36 (8.13)**	**37.43 (8.15)**	**0.049** ^a^	40.3 (3.9)
	LI	43.94 (10.06)	44.91 (11.49)	0.379 ^b^	
IC Pelvic Tilt (deg)	I	16.97 (6.52)	15.44 (6.95)	0.064 ^a^	12.8 (2.3)
	**LI**	**19.4 (6.02)**	**17.66 (6.89)**	**0.047 ^a^**	
Mean Pelvic Tilt (deg)	**-**	20.21 (7.23)	18.89 (7.01)	0.081 ^b^	11.97 (0.56)
ROM Pelvic Tilt (deg)	-	9.29 (3.55)	9.23 (3.95)	0.9 ^b^	1.9 (2.7)

**Table 7:** Kinetics 3D gait outcomes. Based on the data distribution, median/mean (IQR/SD) values are reported. Bolded texts represent statistically significant differences (*p* < 0.05). ^a^ Student *t*-test. I = Impaired; LI = Less Impaired; AF = Ankle Flexion; KF = Knee Flexion; HF = Hip Flexion.

		T0	T1		
Outcome	Impairment Level	Mean/Median (SD/IQR)	Mean/Median (SD/IQR)	*p* Value	Normal Value (SD)
Maximum Moment AF (Nm)	I	0.87 (0.36)	1.1 (0.49)	0.055 ^a^	1.1 (0.2)
	**LI**	**1.01 (0.32)**	**1.08 (0.29)**	**0.035 ^a^**	
Maximum Moment KF (Nm)	I	0.27 (0.18)	0.3 (0.16)	0.312 ^a^	0.48 (0.11)
	LI	0.38 (0.19)	0.33 (0.11)	0.239 ^a^	
Maximum Moment HF (Nm)	I	0.79 (0.2)	0.82 (0.22)	0.649 ^a^	0.8 (0.28)
	LI	0.85 (0.19)	0.88 (0.21)	0.56 ^a^	
Maximum Power AF (W)	I	0.89 (0.4)	0.94 (0.34)	0.458 ^a^	2.34 (0.47)
	LI	1.31 (0.67)	1.42 (0.82)	0.342 ^a^	
Maximum Power KF (W)	**I**	**0.32 (0.17)**	**0.59 (0.38)**	**0.005** ^a^	0.38 (0.28)
	LI	0.38 (0.1)	0.43 (0.24)	0.45 ^a^	
Maximum Power HF (W)	I	0.82 (0.34)	1.1 (0.51)	0.063 ^a^	0.44 (0.3)
	LI	1.14 (0.31)	1.14 (0.41)	0.979 ^a^	

**Table 8:** Gait Variable Scores (GVSs) and Gait Profile Score (GPS). Based on the data distribution, median/mean (IQR/SD) values are reported. Bolded texts represent statistically significant differences (*p* < 0.05). ^a^ Student *t*-test; ^b^ Wilcoxon signed-rank test. I = Impaired; LI = Less Impaired; KFE = Knee Flexion–Extension; HFE = Hip Flexion–Extension.

		T0	T1	
Outcome	Impairment Level	Mean/Median (SD/IQR)	Mean/Median (SD/IQR)	*p* Value
GVS Ankle Dorsiflexion	I	8.49 (2.8)	8.53 (2.96)	0.956 ^a^
	LI	7.89 (2.36)	7.63 (2.7)	0.584 ^a^
GVS Pelvic Tilt	**I**	**7.6 (7.43)**	**6.6 (5.33)**	**0.041** ^b^
	LI	9.14 (3.83)	8.61 (4.42)	0.47 ^a^
GVS HFE	**I**	**9.1 (7.15)**	**7.95 (5.25)**	**0.040** ^b^
	LI	9.8 (4.44)	9.14 (4.67)	0.408 ^a^
GVS KFE	I	11 (10.8)	11.65 (10.05)	0.577 ^b^
	LI	9.57 (5.87)	10.49 (6.07)	0.063 ^a^
GPS	I	12.76 (2.79)	12.47 (2.76)	0.54 ^a^
	LI	11.54 (3.18)	11.6 (3.2)	0.865 ^a^

**Table 9:** L-STIFF and L-FORCE outcome measures. Based on the data distribution, median/mean (IQR/SD) values are reported. Bolded texts represent statistically significant differences (adj *p* < 0.05). ^a^ Student *t*-test; ^b^ Wilcoxon signed-rank test. HF = Hip Flexion; HE = Hip Extension; KF = Knee Flexion; KE = Knee Extension.

			T0	T1	
Outcome		Impairment Level	Mean/Median (SD/IQR)	Mean/Median (SD/IQR)	Adj *p* Value
L-STIFF (Nm/°)	HF 22.5°/s	I	0.31 (0.15)	0.25 (0.11)	0.072 ^a^
		LI	0.29 (0.17)	0.23 (0.13)	0.222 ^b^
	HF 45°/s	**I**	**0.32 (0.12)**	**0.28 (0.12)**	**0.03** **^a^**
		LI	0.32 (0.12)	0.28 (0.08)	0.124 ^a^
	HF 90°/s	**I**	**0.39 (0.15)**	**0.32 (0.1)**	**0.012** **^a^**
		LI	0.35 (0.22)	0.28 (0.12)	0.15 ^b^
	HE 22.5°/s	**I**	**0.25 (0.26)**	**0.26 (0.22)**	**0.045** **^b^**
		LI	0.3 (0.16)	0.24 (0.15)	0.182 ^b^
	HE 45°/s	I	0.36 (0.15)	0.30 (0.14)	0.072 ^a^
		LI	0.35 (0.13)	0.30 (0.1)	0.132 ^a^
	HE 90°/s	**I**	**0.43 (0.18)**	**0.34 (0.14)**	**0.022** **^a^**
		LI	0.38 (0.31)	0.28 (0.15)	0.15 ^b^
	KF 30°/s	I	0.17 (0.09)	0.15 (0.1)	5.11 ^b^
		LI	0.18 (0.13)	0.18 (0.15)	1.96 ^a^
	KF 60°/s	I	0.17 (0.09)	0.18 (0.11)	4.7 ^a^
		LI	0.16 (0.14)	0.14 (0.23)	4.122 ^b^
	KF 120°/s	I	0.24 (0.19)	0.19 (0.18)	2.51 ^b^
		LI	0.23 (0.14)	0.24 (0.18)	2.86 ^a^
	KE 30°/s	I	0.16 (0.09)	0.16 (0.08)	5.2 ^a^
		LI	0.16 (0.1)	0.14 (0.15)	4.2 ^b^
	KE 60°/s	I	0.15 (0.07)	0.17 (0.09)	4.19 ^a^
		LI	0.15 (0.1)	0.14 (0.15)	3.58 ^b^
	KE 120°/s	I	0.21 (0.12)	0.2 (0.11)	5.34 ^a^
		LI	0.19 (0.11)	0.2 (0.15)	5.34 ^a^
L-FORCE (Nm)	HF	I	32.29 (16.03)	38.3 (14.44)	0.345 ^a^
		LI	32.76 (12.76)	33.68 (11.35)	0.717 ^a^
	HE	I	13.46 (15.86)	21.75 (24.84)	0.312 ^b^
		LI	20.69 (15.28)	29.01 (24.32)	0.345 ^a^
	KF	**I**	**10.69 (8.46)**	**13.94 (10.36)**	**0.032** ^a^
		LI	10.83 (21.05)	25.53 (27.32)	0.327 ^b^
	KE	I	17.25 (5.87)	16.21 (5.56)	0.856 ^a^
		LI	23.52 (11.34)	24.36 (11.89)	0.895 ^a^

## Data Availability

The data that support the findings of this study are openly available as of the date of publication on Zenodo at https://doi.org/10.5281/zenodo.15083648 (accessed on 25 March 2025).

## Supplementary Materials

The following supporting information can be downloaded at: https://www.mdpi.com/article/10.3390/s26051438/s1, Table S1: STROBE Statement—checklist of items that should be included in reports of observational studies, Table S2: Gait analysis data in the sagittal, frontal and coronal plane.

## Abbreviations

The following abbreviations are used in this manuscript:

CP	Cerebral palsy
OS	orthopedic surgery
CT	conventional therapy
RAGT	robot-assisted gait training
BWS	body weight support
GMFCS	Gross Motor Function Classification System
6MWT	6 min walking test
GMFM-88	Gross Motor Function Measure-88
FAQ	Gillette Functional Assessment Questionnaire
3DGA	3D-Gait Analysis
GVS	Gait Variable Scores
GPS	Gait Profile Score
I	Impaired
LI	Less Impaired
MCID	minimal clinically important difference
IQR	Interquartile range
SD	standard deviation
ROM	range of motion
TD	typically developing
PROMs	patient-reported outcome measures
COPM	Canadian Occupational Performance Measure
GAS	Goal Attainment Scaling

## References

[1] Rosenbaum P., Paneth N., Leviton A., Goldstein M., Bax M. (2007). A report: The definition and classification of cerebral palsy April 2006. Dev. Med. Child Neurol..

[2] Graham H.K., Rosenbaum P., Paneth N., Dan B., Lin J.-P., Damiano D.L., Becher J.G., Gaebler-Spira D., Colver A., Reddihough D.S. (2016). Cerebral palsy. Nat. Rev. Dis. Prim..

[3] Clayton-Krasinski D., Fieback L., Cameron M.H., Monroe L.G. (2007). Chapter 15—Pediatric Nonprogressive Central Nervous System Disorders. Physical Rehabilitation.

[4] Graham H.K., Selber P. (2003). Musculoskeletal aspects of cerebral palsy. J. Bone Jt. Surg..

[5] Novak I., Morgan C., Adde L., Blackman J., Boyd R.N., Brunstrom-Hernandez J., Cioni G., Damiano D., Darrah J., Eliasson A.C. (2017). Early, accurate diagnosis and early intervention in cerebral palsy: Advances in diagnosis and treatment. JAMA Pediatr..

[6] McGinley J.L., Dobson F., Ganeshalingam R., Shore B.J., Rutz E., Graham H.K. (2012). Single-event multilevel surgery for children with cerebral palsy: A systematic review. Dev. Med. Child Neurol..

[7] Galey S.A., Lerner Z.F., Bulea T.C., Zimbler S., Damiano D.L. (2017). Effectiveness of surgical and non-surgical management of crouch gait in cerebral palsy: A systematic review. Gait Posture.

[8] Patikas D., Wolf S.I., Armbrust P., Mund K., Schuster W., Dreher T., Döderlein L. (2006). Effects of a postoperative resistive exercise program on the knee extension and flexion torque in children with cerebral palsy: A randomized clinical trial. Arch. Phys. Med. Rehabil..

[9] Seniorou M., Thompson N., Harrington M., Theologis T. (2007). Recovery of muscle strength following multi-level orthopaedic surgery in diplegic cerebral palsy. Gait Posture.

[10] Sharma N., Classen J., Cohen L.G. (2013). Neural Plasticity and Its Contribution to Functional Recovery.

[11] De Luca R., Bonanno M., Settimo C., Muratore R., Calabrò R.S. (2022). Improvement of Gait after Robotic-Assisted Training in Children with Cerebral Palsy: Are We Heading in the Right Direction?. Med. Sci..

[12] Zhong B., Niu W., Broadbent E., McDaid A., Lee T.M.C., Zhang M. (2019). Bringing Psychological Strategies to Robot-Assisted Physiotherapy for Enhanced Treatment Efficacy. Front. Neurosci..

[13] Llamas-Ramos R., Sánchez-González J.L., Llamas-Ramos I. (2022). Robotic Systems for the Physiotherapy Treatment of Children with Cerebral Palsy: A Systematic Review. Int. J. Environ. Res. Public Health.

[14] Chen H., Yun G., Wang J., Fan Y., Zhao W., Zhan Y., Sun S., Wang Y. (2026). Robot-assisted gait training for lower limb motor recovery in cerebral palsy: A meta-analysis of combined and standalone approaches. Gait Posture.

[15] Lefmann S., Russo R., Hillier S. (2017). The effectiveness of robotic-assisted gait training for paediatric gait disorders: Systematic review. J. Neuroeng. Rehabil..

[16] Mataki Y., Kamada H., Mutsuzaki H., Shimizu Y., Takeuchi R., Mizukami M., Yoshikawa K., Takahashi K., Matsuda M., Iwasaki N. (2018). Use of Hybrid Assistive Limb (HAL^®^) for a postoperative patient with cerebral palsy: A case report. BMC Res. Notes.

[17] Kuroda M.M., Mutsuzaki H., Nakagawa S., Yoshikawa K., Takahashi K., Mataki Y., Takeuchi R., Iwasaki N., Yamazaki M. (2022). Short-Term Outcome of Rehabilitation Program with Hybrid Assistive Limb after Tendon Lengthening in Patients with Cerebral Palsy. Pediatr. Rep..

[18] Gorton G.E., Abel M.F., Oeffinger D.J., Bagley A., Rogers S.P., Damiano D., Romness M., Tylkowski C. (2009). A prospective cohort study of the effects of lower extremity orthopaedic surgery on outcome measures in ambulatory children with cerebral palsy. J. Pediatr. Orthop..

[19] von Elm E., Altman D.G., Egger M., Pocock S.J., Gøtzsche P.C., Vandenbroucke J.P. (2008). The Strengthening the Reporting of Observational Studies in Epidemiology (STROBE) statement: Guidelines for reporting observational studies. J. Clin. Epidemiol..

[20] Demont A., Gedda M., Lager C., de Lattre C., Gary Y., Keroulle E., Feuillerat B., Caudan H., Sancelme Z., Isapof A. (2022). Evidence-Based, Implementable Motor Rehabilitation Guidelines for Individuals With Cerebral Palsy. Neurology.

[21] Palisano R., Rosenbaum P., Walter S., Russell D., Wood E., Galuppi B. (1997). Development and reliability of a system to classify gross motor function in children with cerebral palsy. Dev. Med. Child Neurol..

[22] ATS Committee (2002). ATS statement: Guidelines for the six-minute walk test. Am. J. Respir. Crit. Care Med..

[23] Russell D.J., Rosenbaum P.L., Cadman D.T., Gowland C., Hardy S., Jarvis S. (1989). The gross motor function measure: A means to evaluate the effects of physical therapy. Dev. Med. Child Neurol..

[24] Novacheck T.F., Stout J.L., Tervo R. (2000). Reliability and validity of the Gillette Functional Assessment Questionnaire as an outcome measure in children with walking disabilities. J. Pediatr. Orthop..

[25] Davis R.B., Ounpuu S., Tyburski D., Gage J.R. (1991). Davis_1991.pdf. Hum. Mov. Sci..

[26] Firth G.B., Passmore E., Sangeux M., Thomason P., Rodda J., Donath S., Selber P., Graham K.H. (2013). Multilevel surgery for equinus gait in children with spastic diplegic cerebral palsy medium-term follow-up with gait analysis. J. Bone Jt. Surg..

[27] Druzbicki M., Rusek W., Snela S., Dudek J., Szczepanik M., Zak E., Durmala J., Czernuszenko A., Bonikowski M., Sobota G. (2013). Functional effects of robotic-assisted locomotor treadmill therapy in children with cerebral palsy. J. Rehabil. Med..

[28] Wallard L., Dietrich G., Kerlirzin Y., Bredin J. (2017). Robotic-assisted gait training improves walking abilities in diplegic children with cerebral palsy. Eur. J. Paediatr. Neurol..

[29] Wallard L., Dietrich G., Kerlirzin Y., Bredin J. (2018). Effect of robotic-assisted gait rehabilitation on dynamic equilibrium control in the gait of children with cerebral palsy. Gait Posture.

[30] Aycardi L.F., Cifuentes C.A., Múnera M., Bayón C., Ramírez O., Lerma S., Frizera A., Rocon E. (2019). Evaluation of biomechanical gait parameters of patients with Cerebral Palsy at three different levels of gait assistance using the CPWalker. J. Neuroeng. Rehabil..

[31] Storm F.A., Petrarca M., Beretta E., Strazzer S., Piccinini L., Maghini C., Panzeri D., Corbetta C., Morganti R., Reni G. (2020). Minimum Clinically Important Difference of Gross Motor Function and Gait Endurance in Children with Motor Impairment: A Comparison of Distribution-Based Approaches. Biomed Res. Int..

[32] Oeffinger D., Bagley A., Rogers S., Gorton G., Kryscio R., Abel M., Damiano D., Barnes D., Tylkowski C. (2008). Outcome tools used for ambulatory children with cerebral palsy: Responsiveness and minimum clinically important differences. Dev. Med. Child Neurol..

[33] Flux E., van der Krogt M.M., Cappa P., Petrarca M., Desloovere K., Harlaar J. (2020). The Human Body Model versus conventional gait models for kinematic gait analysis in children with cerebral palsy. Hum. Mov. Sci..

[34] Beretta E., Storm F.A., Strazzer S., Frascarelli F., Petrarca M., Colazza A., Cordone G., Biffi E., Morganti R., Maghini C. (2020). Effect of Robot-Assisted Gait Training in a Large Population of Children With Motor Impairment Due to Cerebral Palsy or Acquired Brain Injury. Arch. Phys. Med. Rehabil..

[35] Kim C.J., Son S.M. (2014). Comparison of spatiotemporal gait parameters between children with normal development and children with diplegic cerebral palsy. J. Phys. Ther. Sci..

[36] Wang Y., Zhang P., Li C. (2023). Systematic review and network meta-analysis of robot-assisted gait training on lower limb function in patients with cerebral palsy. Neurol. Sci..

[37] Hunt M., Everaert L., Brown M., Muraru L., Hatzidimitriadou E., Desloovere K. (2022). Effectiveness of robotic exoskeletons for improving gait in children with cerebral palsy: A systematic review. Gait Posture.

[38] Yngve D.A. (2021). Recurvatum of the Knee in Cerebral Palsy: A Review. Cureus.

[39] Bell K.J., Õunpuu S., DeLuca P.A., Romness M.J. (2002). Natural progression of gait in children with cerebral palsy. J. Pediatr. Orthop..

[40] van der Krogt M.M., Sloot L.H., Buizer A.I., Harlaar J. (2015). Kinetic comparison of walking on a treadmill versus over ground in children with cerebral palsy. J. Biomech..

[41] Schmartz A.C., Meyer-Heim A.D., Müller R., Bolliger M. (2011). Measurement of muscle stiffness using robotic assisted gait orthosis in children with cerebral palsy: A proof of concept. Disabil. Rehabil. Assist. Technol..

[42] Saleh E., Dahan-Oliel N., Montpetit K., Benaroch T., Yap R., Barakat N., Mulcahey M.J. (2018). Functional Gains in Children With Spastic Hemiplegia Following a Tendon Achilles Lengthening Using Computerized Adaptive Testing—A Pilot Study. Child Neurol. Open.

[43] Grecco L.A.C., De Freitas T.B., Satie J., Bagne E., Oliveira C.S., De Souza D.R. (2013). Treadmill training following orthopedic surgery in lower limbs of children with cerebral palsy. Pediatr. Phys. Ther..

[44] Carman S., Wall S., Stewart K., Mudge A., Axt M. (2025). Lower limb orthopedic surgery in children and adolescents with cerebral palsy is well captured using individualized Goal Attainment Scale (GAS) and Canadian Occupational Performance Measure (COPM) goals. Disabil. Rehabil..

